# High Prevalence of *A*^−^*β*^+^ Ketosis-Prone Diabetes in Children with Type 2 Diabetes and Diabetic Ketoacidosis at Diagnosis: Evidence from the Rare and Atypical Diabetes Network (RADIANT)

**DOI:** 10.1155/2024/5907924

**Published:** 2024-03-04

**Authors:** Elizabeth Kubota-Mishra, Xiaofan Huang, Charles G. Minard, Marcela Astudillo, Ahmad Refaey, Graciela Montes, Stephanie Sisley, Nalini Ram, William E. Winter, Rochelle N. Naylor, Ashok Balasubramanyam, Maria J. Redondo, Mustafa Tosur, RADIANT Study Group

**Affiliations:** 1 Department of Pediatrics, The Division of Diabetes and Endocrinology, Baylor College of Medicine, Texas Children’s Hospital, Houston, TX, USA; 2 Institute for Clinical and Translational Research, Baylor College of Medicine, Houston, TX, USA; 3 University of Houston, Houston, TX, USA; 4 Division of Diabetes, Endocrinology and Metabolism, Baylor College of Medicine, Houston, TX, USA; 5 USDA/ARS Children’s Nutrition Research Center, Houston, TX, USA; 6 Department of Pathology, University of Florida, Gainesville, FL, USA; 7 Section of Adult and Pediatric Endocrinology, Diabetes and Metabolism, Departments of Pediatric and Medicine, University of Chicago, Chicago, IL, USA; 8 The RADIANT Data Coordinating Center, Health Informatics Institute, University of South Florida, Tampa, FL, USA

## Abstract

**Background.:**

*A*^−^*β*^+^ ketosis-prone diabetes (KPD) in adults is characterized by presentation with diabetic ketoacidosis (DKA), negative islet autoantibodies, and preserved *β*-cell function in persons with a phenotype of obesity-associated type 2 diabetes (T2D). The prevalence of KPD has not been evaluated in children. We investigated children with DKA at “T2D” onset and determined the prevalence and characteristics of pediatric *A*^−^*β*^+^ KPD within this cohort.

**Methods.:**

We reviewed the records of 716 children with T2D at a large academic hospital and compared clinical characteristics of those with and without DKA at onset. In the latter group, we identified patients with *A*^−^*β*^+^ KPD using criteria of the Rare and Atypical Diabetes Network (RADIANT) and defined its prevalence and characteristics.

**Results.:**

Mean age at diagnosis was 13.7 ± 2.4 years: 63% female; 59% Hispanic, 29% African American, 9% non-Hispanic White, and 3% other. Fifty-six (7.8%) presented with DKA at diagnosis and lacked islet autoantibodies. Children presenting with DKA were older and had lower C-peptide and higher glucose concentrations than those without DKA. Twenty-five children with DKA (45%) met RADIANT *A*^−^*β*^+^ KPD criteria. They were predominantly male (64%), African American or Hispanic (96%), with substantial C-peptide (1.3 ± 0.7 ng/mL) at presentation with DKA and excellent long-term glycemic control (HbA1c 6.6% ± 1.9% at follow-up (median 1.3 years postdiagnosis)).

**Conclusions.:**

In children with a clinical phenotype of T2D and DKA at diagnosis, approximately half meet criteria for *A*^−^*β*^+^ KPD. They manifest the key characteristics of obesity, preserved *β*-cell function, male predominance, and potential to discontinue insulin therapy, similar to adults with *A*^−^*β*^+^ KPD.

## 1. Introduction

The incidence of pediatric diabetes is increasing in parallel with the rising epidemic of pediatric obesity [1]. The current classification system for pediatric diabetes mellitus, in which children are primarily defined as having either “type 1” (T1D) or “type 2” diabetes (T2D) [2] fails to capture the diverse clinical presentations, natural histories, and treatment responses of a large proportion of patients.

Ketosis-prone diabetes (KPD) is a heterogeneous, emerging syndrome characterized by diabetic ketoacidosis (DKA) in patients who lack a typical phenotype of autoimmune T1D [3]. KPD is most accurately categorized by a validated “*Aβ*” classification system that predicts the natural history of KPD patients with regard to long-term *β*-cell function, glycemic control, and requirement for insulin therapy. This classification system defines four KPD subgroups based on islet autoantibody status (“*A*^+^” or “*A*^−^”) and evidence of *β*-cell functional reserve (“*β*^+^” or “*β*^−^”) [3–5]. It has been utilized to demonstrate distinct pathophysiologic mechanisms and clinically significant outcomes specific to each subgroup in large, multiethnic cohorts of adult patients presenting with DKA [4, 5]. Of particular relevance to the present investigation, adults with *A*^−^*β*^+^ KPD are characterized by late-onset diabetes, obesity, negative islet autoantibodies, and preserved *β*-cell function despite presentation with DKA. In addition, individuals with this atypical form of diabetes are able to discontinue insulin therapy within 4–12 weeks following the index episode of DKA and maintain excellent, long-term glycemic control on treatment with oral agents alone [4, 6].

In the SEARCH for Diabetes in Youth study, approximately 10% of pediatric patients with new onset T2D were found to present with DKA [7]. Among the children who presented with DKA at diagnosis, children with obesity were older compared to their lean counterparts, and in a U.S. cohort of children with non-insulin-dependent diabetes, those presenting with DKA were more likely to be male and African American and have acanthosis nigricans [8, 9]. Despite presenting with DKA, up to half of the children with obesity who presented with DKA at initial diagnosis of diabetes successfully discontinued insulin therapy within a median period of 1.25 months [8]. These data suggest that a substantial number of youth with obesity and newly diagnosed diabetes may have the unique features of the *A*^−^*β*^+^ subgroup of KPD.

The Rare and Atypical Diabetes Network (RADIANT) is a consortium of universities, hospitals, and clinics in the United States dedicated to identifying, studying, and classifying atypical forms of diabetes [10]. *A*^−^*β*^+^ KPD is included among prespecified phenotypes of atypical diabetes of interest to the RADIANT investigators. We and others have previously reported extensively on the validated diagnostic criteria, characteristics, and natural history of adult patients with *A*^*−*^*β*^+^ KPD [3–5, 11–13]. There are very sparse data on this syndrome in pediatric populations. Lack of awareness of this emerging, atypical condition among pediatricians is reflected in the fact that most children with overweight or obesity presenting with DKA at initial diagnosis are labeled as having either “T1D” or “T2D” without further elaboration [7]. To better understand the prevalence and characteristics of pediatric *A*^−^*β*^+^ KPD, we first explored the clinical characteristics of children with a phenotype of T2D who presented with DKA at initial diagnosis compared to children with T2D who did not present with DKA. Then, we applied the diagnostic criteria adopted by RADIANT to circumscribe and describe patients with *A*^*−*^*β*^+^ KPD among those who presented with DKA.

## 2. Methods

### 2.1. Participants.

**Figure 1:**
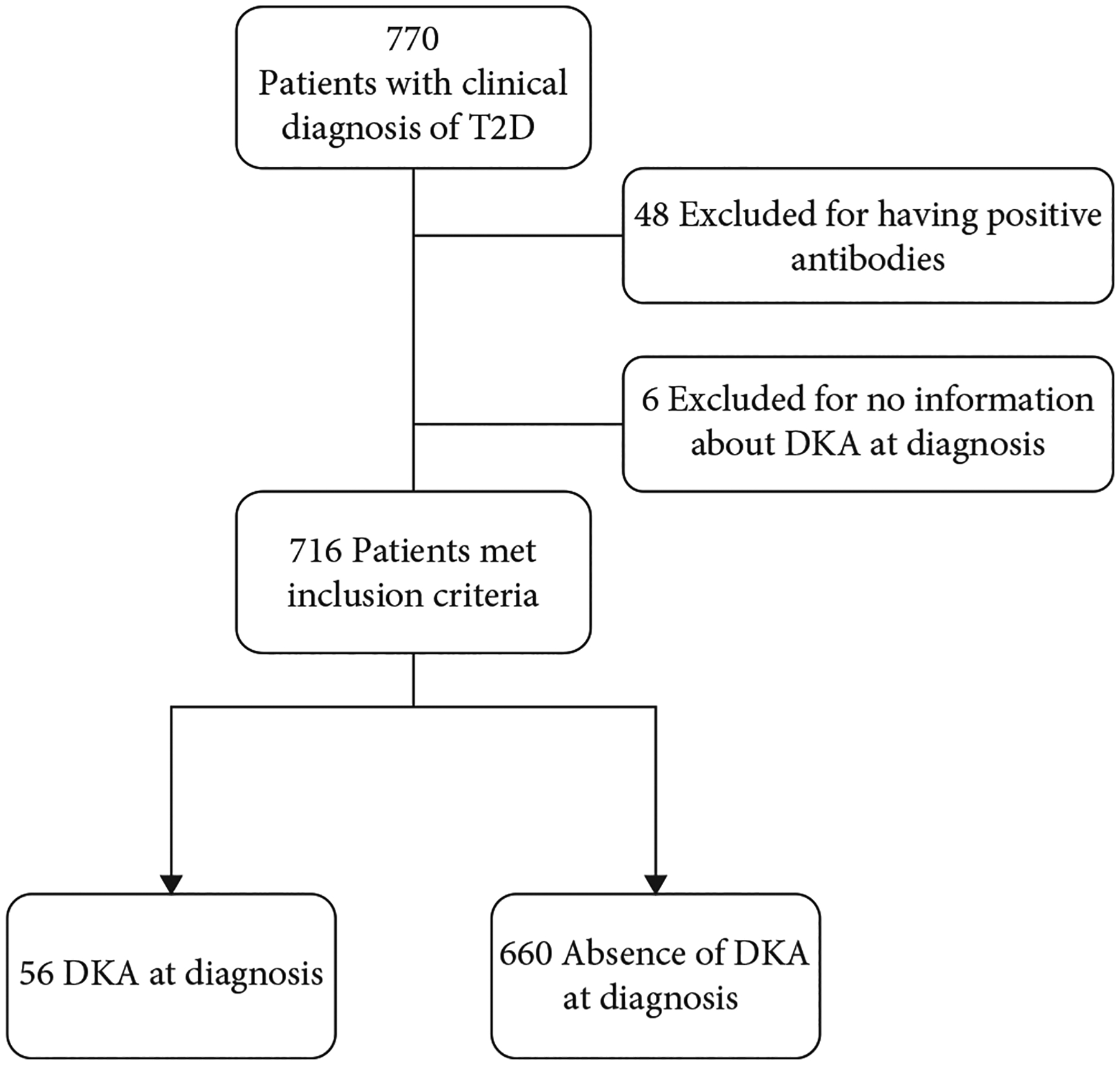
CONSORT diagram of eligibility.

We performed a retrospective electronic medical record (EMR) review of 770 pediatric patients (<19 years old) seen at Texas Children’s Hospital (Houston, TX) with a diagnosis of T2D between July 2016 and July 2019. The study cohort included 716 participants after excluding those who were positive for any islet autoantibody (*n* = 48) and those with inadequate information to confirm DKA at diabetes diagnosis (*n* = 6; Figure 1). Diagnosis of “T2D” was determined by a medical provider, based on standard clinical and laboratory characteristics [14, 15]. Of 716 subjects, 87.8% (*n* = 629) had one or more islet autoantibodies measured (directed against the 65 kDa glutamic acid decarboxylase (GAD-65), insulinoma associated antigen-512 (ICA-512), zinc transporter-8 (ZnT8), and/or insulin (IAA) via Quest Diagnostics Nichols Institute (San Juan Capistrano, CA, USA)). The remaining 87 subjects (12.2%) were not tested for islet antibodies. Among those who were tested for at least one islet autoantibody (*n* = 629), 596 (94.8%) were negative for three or more islet autoantibodies (the combination of GAD65-Ab, ICA512-Ab, and ZnT8-Ab or GAD65-Ab, ICA512-Ab, and IAA), 18 (2.9%) were negative for one or two islet autoantibodies (GAD65-Ab and ICA512-Ab, GAD65-Ab alone, ICA512-Ab alone, or IAA alone), and the remaining 15 (2.4%) were negative for GAD65-Ab and ICA512-Ab but were positive for IAA while on insulin therapy. Of note, because the development of IAA is a known consequence of insulin therapy, positive IAA results after insulin initiation were disregarded and those subjects were not excluded from the study. Of the 629 patients tested for islet antibodies, 435 (69.2%) had serum collected for antibody measurement within the first 7 days after diagnosis.

The study was approved by the BCM Institutional Review Board (IBR; H-45325), which waived the need for informed consent.

### 2.2. Data Collection.

We identified children with *A*^−^*β*^+^ KPD based on validated criteria [3–5] adapted by RADIANT, which are: (1) episode of DKA at initial diagnosis of diabetes or within 6 months of diagnosis; (2) not being treated with a sodium–glucose cotransporter-2 (SGLT2) inhibitor; (3) absence of islet autoantibodies measured within 3 years after diagnosis; (4) achieving hemoglobin A1c (HbA1c) ≤7.5% postdiagnosis and meeting at least one of the following criteria: (i) insulin therapy discontinued within 2 years after presentation with DKA, (ii) total daily insulin requirement <0.5 units/kg/day within 6 months after presentation with DKA, or (iii) fasting C-peptide level >1ng/mL (or random >1.5 ng/mL). Random C-peptide levels were measured within 9 months of the index DKA episode. The fasting C-peptide cutoff to distinguish “*β*^+^” from “*β*^−^” status in KPD patients was previously established using the receiver-operator characteristic area under the curve (ROC-AUC) analysis [4].

The following demographic and clinical data were collected from the patients’ medical records: age at diagnosis, sex, race/ethnicity, body mass index (BMI) percentiles, and BMI *z*-score, DKA at diagnosis (defined by the International Society for Pediatric and Adolescent Diabetes [16]), occurrence of DKA at any time, acanthosis nigricans at diagnosis, tanner stage at diagnosis, presence of nonalcoholic fatty liver disease (NAFLD; per clinical diagnosis in the EMR), presence of polycystic ovary syndrome (PCOS; per clinical diagnosis in the EMR), treatment regimen, presence of hypertension, systolic blood pressure at diagnosis, diastolic blood pressure at diagnosis, presence of diabetic retinopathy and microalbuminuria (spot collection with two of three samples showing >30 *μ*g/mg creatinine [17]) within the first year after diagnosis, and family history of diabetes mellitus. The race and ethnicity categorizations were based on self-report per documentation in the EMR. We used the following racial/ethnic categories: Hispanic, non-Hispanic White, African American, and other races. The following laboratory data were also collected; random C-peptide at diagnosis (measured via Abbott ARCHITECT C-peptide chemiluminescent microparticle immunoassay (CMIA)), glucose at diagnosis (either serum or point of care), HbA1c at diagnosis (either laboratory (measured by end-point) or point of care), serum titers of GAD65-Ab, ICA512-Ab, ZnT8-Ab, and IAA-Ab; lipid profile; and HbA1c at the last office visit. Laboratory HbA1c and point of care HbA1c levels were measured using whole blood immunoassay on the Vitros 5600 and DCA Vantage Analyzer, respectively, and both were well-correlated (interassay coefficient <3%) to NGSP (National Glycohemoglobin Standardization Program). Patient records were also reviewed for evidence of acute pancreatitis (as defined by Banks et al. [18]) or an acute infection at the time initial presentation, to assess whether the DKA episode at the time of diabetes diagnosis was secondary to a clinically identifiable acute precipitating factor (“provoked” DKA) or not associated with such a precipitating factor (“unprovoked” DKA).

### 2.3. Statistics and Data Analysis.

The patient characteristics are presented as median with 25^th^ and 75^th^ percentile levels, mean with standard deviation, or frequency with proportion. Summary statistics were stratified by presence or absence of DKA at T2D diagnosis and compared using the Wilcoxon rank sum test or the Pearson *χ*^2^ test. Univariable logistic regression was used to identify the baseline characteristics that were significantly associated with diagnosis of DKA. Multiple logistic regression was used to include all the significant factors from the univariable model, and stepwise selection was used to choose the best reduced model by Akaike information criterion. Among patients who had DKA at T2D diagnosis, a *t*-test, Wilcoxon rank sum test, the Pearson *χ*^2^ test, or Fisher’s exact test were used, as appropriate, to compare those who met *A*^−^*β*^+^ KPD criteria vs. those who did not. A significance level of 0.05 was used. All analyses were conducted using *R* statistical software (RStudio Team (2022), RStudio: Integrated Development for R. RStudio, PBC, Boston, MA; URL http://www.rstudio.com/).

## 3. Results

We studied 716 children with T2D evaluated during a 3-year study period at Texas Children’s Hospital. Mean age at diagnosis was 13.7 2.4 years; 63% were female; 59% Hispanic, 29% African American, 9% non-Hispanic White, and 3% other races. Fifty-six (7.8%) presented with DKA at diagnosis and lacked serum islet autoantibodies. Baseline characteristics are summarized in Table 1.

African American race (45% vs. 28%, *p* = 0.006) and male sex (61% vs. 35%, *p*≤0.001) were more frequent in those presenting with DKA compared to those without DKA. In addition, children with DKA at diagnosis were older and more likely to have lower random C-peptide, higher glucose, and higher HbA1c at diagnosis than those without DKA at diagnosis (Table 1). There were no differences between the two groups in the frequency of comorbidities (i.e., hypertension, dyslipidemia, NAFLD, PCOS, microalbuminuria within 1 year of diagnosis and retinopathy within 1 year of diagnosis). Of note, only two patients in our cohort had retinopathy, and they did not have DKA at diagnosis.

In a multivariable model using age, sex, race, C-peptide, glucose, HbA1c, and overweight/obesity at diagnosis as covariates, older age, lower C-peptide, and higher glucose at diagnosis remained significantly associated with DKA (Table 2). Although overweight/obesity was not significant in this multivariable model, when BMI ***z***-score was included as a continuous variable, instead of overweight/obesity as a categorical variable, it was significantly associated with DKA (*p* = 0.004).

Twenty-five children with DKA (45%) met criteria for *A*^−^*β*^+^ KPD (Table 3). Two patients had a clinically significant stressful event that could have precipitated the episode of DKA and 20 lacked such an event preceding the episode of DKA (“unprovoked DKA”). Documentation of the presence or absence of any precipitating factors for DKA was lacking for three patients who did not present to our hospital during the initial episode of DKA. Fifty-five percent did not meet the KPD criteria; the predominant reason, 80%, for their exclusion was that insulin therapy was continued at a dose higher than 0.5 units/kg/day for more than 2 years after the index episode of DKA. The remaining 20% of patients who did not meet the KPD criteria were lost to follow-up; so, we were unable to assess their long-term insulin needs. Of the 25 children with A^*−*^*β*^+^ KPD who met all RADIANT criteria, the mean age at diagnosis was 14.9 ± 2 years. The group was 64% male; 56% Hispanic and 40% African American. Mean HbA1c at diagnosis was 12.2% ± 1.2%, but the majority had excellent glycemic control at their last office visit with a mean HbA1c of 6.6% ± 1.9% (median 1.3 years after diagnosis; Table 4). Insulin was safely discontinued in 18 of these patients without affecting their ability to achieve good glycemic control. Of the seven patients who continued to receive insulin treatment, at the last office visit, six required <0.5 units/kg/day and one required 1.2 units/kg/day for their daily insulin requirement but maintained a high random C-peptide. Two patients had hypertension, PCOS, or NAFLD; and 22 patients (88%) were diagnosed with dyslipidemia within 1 year of diagnosis. Of the identified pediatric *A*^−^*β*^+^ KPD patients, 24% (*n* = 6) agreed to participate in RADIANT.

Among the T2D patients presenting with DKA, those who met *A*^−^*β*^+^ KPD criteria had a significantly lower HbA1c during follow-up than those who did not. There was also a trend (*p* = 0.08) toward a higher proportion of unprovoked DKA in the former group (Table 4).

## 4. Discussion

We found that among racially/ethnically diverse children with a phenotype of “T2D” who presented with DKA at initial diagnosis of diabetes, approximately 50% met the criteria for *A*^*−*^*β*^+^ KPD and almost all belonged to Hispanic or African American race/ethnicity groups. The clinical and biochemical characteristics of pediatric *A*^*−*^*β*^+^ KPD are strikingly similar to those of adults with this syndrome, including the predilection for African American and Hispanic race/ethnicity, evidence of preserved *β*-cell function even at the time of the index DKA episode, rapid near normalization of glycemic control leading to insulin independence, and male predominance [3–5, 11, 13, 19]. Recognition of this high prevalence in children with diabetes is important because of the unique natural history, pathogenesis, and potential for insulin-independence in patients with *A*^−^*β*^+^ KPD.

Our pediatric cohort with *A*^*−*^*β*^+^ KPD demonstrated a substantial serum C-peptide level at the time of the index DKA episode, similar to levels reported in *A*^−^*β*^+^ KPD adults at the time of acute presentation with DKA [19] and much higher than the levels observed in pediatric patients with typical autoimmune T1D with DKA at diagnosis [20]. Jahoor et al. [21] reported that at the time of acute presentation with DKA, adults with KPD had a mean serum C-peptide level of 1.1 ± 0.2 ng/mL, whereas adults with autoimmune T1D had a mean C-peptide level of 0.34 ± 0.1 ng/mL [22]. A random serum C-peptide level obtained at the time of the initial DKA episode may be a useful biomarker to identify children with potential *A*^−^*β*^+^ KPD, and prospective studies to establish diagnostic cut-offs would be an important follow-up to the present report.

A majority of the children with *A*^*−*^*β*^+^ KPD were males of African American and Hispanic race/ethnicity, which is in contrast to the female predominance among children with T2D, including subgroups of children belonging to racial/ethnic groups underrepresented in medicine (African American, Asian-Pacific Islander, Native American, and Hispanic) [23]. Numerous studies have noted a male predominance in adults with *A*^−^*β*^+^ KPD [4, 13, 19], specifically among adults with unprovoked *A*^*−*^*β*^+^ KPD [19] the majority of whom remain insulin-independent with good glycemic control for a median period of over 4 years [4, 13, 24]. In contrast, adults with provoked *A*^*−*^*β*^+^ KPD (i.e., those presenting with DKA associated with a clinically significant precipitating factor) lack male predominance, have a higher frequency of family history of diabetes, are less likely to have obesity or be African American, and relapse to insulin-dependence with *β*-cell function decline sooner than those with unprovoked *A*^*−*^*β*^+^ KPD [19]. There is also an important distinction in pathophysiology between adults with unprovoked *A*^−^*β*^+^ KPD compared to those with provoked *A*^−^*β*^+^ KPD; the former lack evidence for both humoral (autoantibodies) and cellular (T cell–mediated) islet autoimmunity and have a higher frequency of an HLA allele that is protective against autoimmune T1D (DQB1 *0602), whereas the latter have a high frequency of T cell–mediated islet autoimmunity and are more likely to possess an HLA allele associated with susceptibility to T1D (DQB1 *0302). Thus, islet autoimmunity not manifested by traditional T1D autoantibodies appears to play a role in the progressive decline of *β*-cell function in provoked *A*^−^*β*^+^ KPD [25], whereas unique metabolic factors may be responsible for the proclivity to develop DKA among patients with unprovoked *A*^*−*^*β*^+^ KPD. Because very few in our pediatric cohort of *A*^−^*β*^+^ KPD had provoked DKA (two patients out of 25), we were restricted in the ability to analyze the characteristics of provoked *A*^−^*β*^+^ KPD in children.

We previously showed that adults with unprovoked *A*^*−*^*β*^+^ KPD have unique fasting plasma metabolite signatures, including decreased levels of the leucine catabolite isovaleryl carnitine and of tricarboxylic acid cycle (TCA) intermediates, together with higher glutamate but lower glutamine and citrulline compared with obese, nondiabetic controls [12]. These findings led to focused kinetic studies using stable isotope infusions and mass spectrometry that indicated their propensity to develop DKA, may be due to excessive production of ketones from accelerated leucine catabolism associated with impaired ketone oxidation due to slowing of the TCA cycle [12]. Metabolomics and kinetic studies in unprovoked *A*^−^*β*^+^ KPD patients also demonstrated significantly decreased endogenous arginine availability in response to hyperglycemia compared to controls with obesity; this was associated with a marked defect in insulin secretion in response to glucose but normal insulin secretion in response to arginine [26]. Studies of West African KPD patients with a likely *A*^*−*^*β*^+^ phenotype have suggested a role for human herpesvirus 8 in the pathogenesis [27], and a higher prevalence of glucose-6-phosphate dehydrogenase (G6PD) deficiency correlated with insulin deficiency in these patients [28]. The pathogenesis of this syndrome in pediatric patients remains to be elucidated and is a focus of investigations in the RADIANT study.

Pinhas-Hamiel et al. [9] studied 42 adolescents with T2D who were negative for islet cell antibodies and noted that seven presented with DKA and five with ketosis. It is likely that many of these patients fulfilled criteria for *A*^*−*^*β*^+^ KPD, given that they were overweight or had obesity at presentation and could discontinue insulin therapy with good glycemic control on metformin. It will be important to perform prospective studies to delineate the factors that differentiate children with *A*^*−*^*β*^+^ KPD who have a prolonged insulin-independent course from those who have a more rapid decline of *β*-cell function and relapse to insulin-dependence.

**Figure 2:**
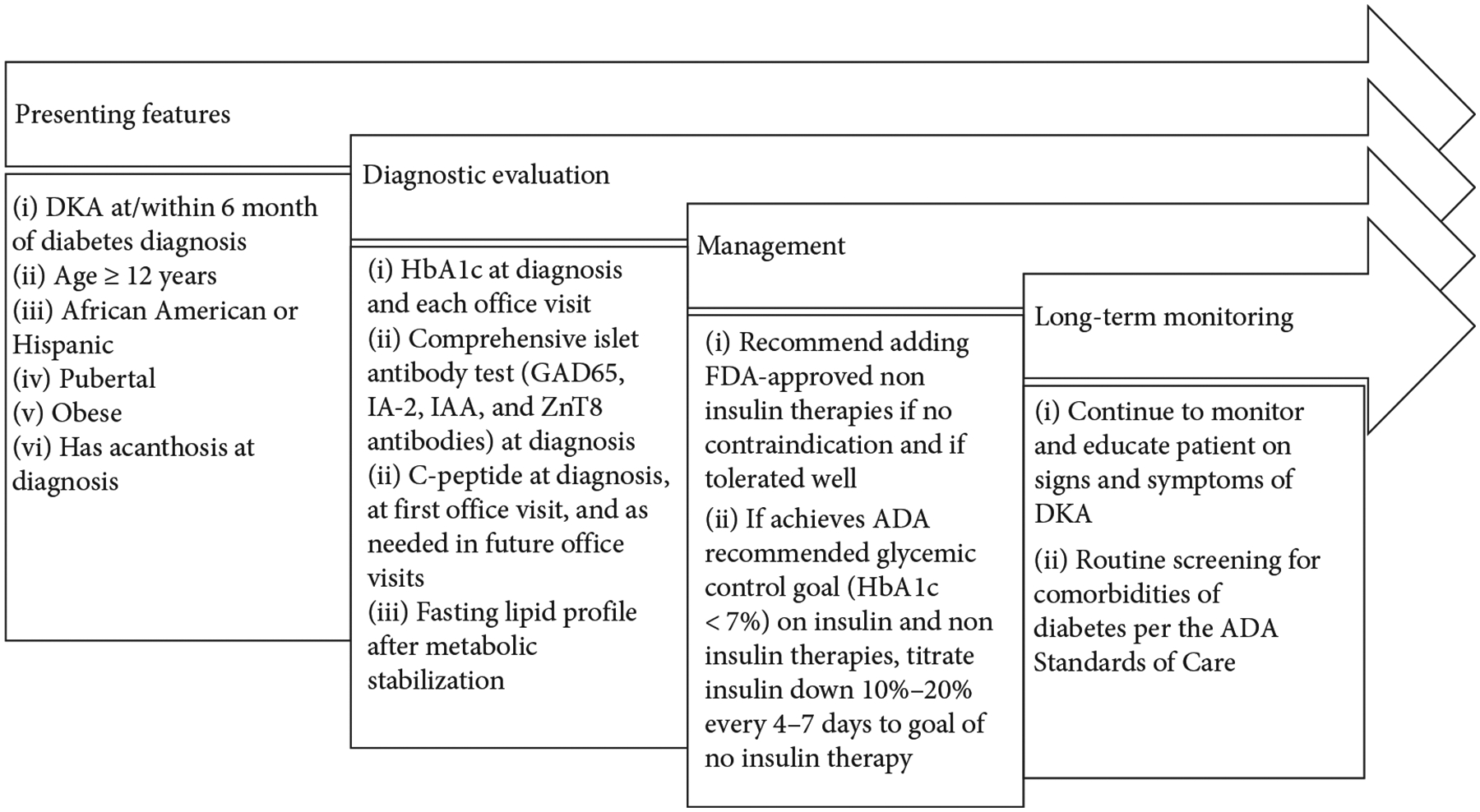
Diagnostic and management recommendations for pediatric patients with *A*^−^*β*^+^ KPD.

A clinically significant aspect of identifying children with *A*^*−*^*β*^+^ KPD shortly after the index episode of DKA is the fact that insulin therapy may be safely discontinued with maintenance of excellent metabolic control with oral agents alone, in the majority of these patients. Although there is no widely accepted protocol to safely discontinue insulin therapy in children with *A*^−^*β*^+^ KPD, clinicians may consider decreasing the insulin doses gradually (10%–20% decrease at intervals of 4–7 days) with close monitoring of blood glucose levels during this transition (Figure 2). However, both because of the reluctance of pediatric endocrinologists to withhold insulin therapy in children who have experienced an episode of DKA, and due to limited availability of FDA-approved non-insulin treatment options in pediatrics, insulin is often continued for long periods of time in these patients. This, in addition to potential pathophysiologic differences between pediatric and adult patients with KPD, may account for the higher rate of insulin discontinuation in adults with *A*^−^*β*^+^ KPD than in the children we identified in the present study [24].

In the cohort of T2D patients presenting with DKA, comparison of the clinical characteristics of those who met *A*^−^*β*^+^ KPD criteria versus those who did not revealed some interesting differences. Those in the former group had significantly better glycemic control during follow-up this is perhaps not surprising as attainment of good glycemic control is itself an element in the prespecified criteria. The former group also showed a strong trend toward a higher frequency of unprovoked DKA, suggesting that our criteria to define *A*^*−*^*β*^+^ KPD in children captures a singular characteristic of this condition in adults, i.e., the development of DKA without a notable precipitating factor in patients with an apparent phenotype of T2D. This is the first pediatric study reporting the prevalence of *A*^*−*^*β*^+^ KPD in a large and ethnically diverse patient cohort with comprehensive clinical characterization, including data on islet autoantibodies and C-peptide measurements at diagnosis. Limitations of the study include its retrospective design and some missing data which limited the full description and capture of patients with KPD. In addition, it cannot be confirmed that all subjects had islet autoantibody testing at disease onset. Because all the patients presented with DKA prior to the onset of the COVID-19 pandemic, our data does not reflect the effect of SARS-CoV2 infection on the occurrence of DKA or features of the clinical course shortly following the DKA episode. A recent study demonstrated that COVID-19 illness may cause a unique variant of provoked *A*^−^*β*^+^ KPD [29, 30], and it would be of great interest to identify such a syndrome in children who develop new-onset diabetes with DKA at presentation following infection with SARS-CoV2.

In conclusion, we found that among multiethnic children presenting to a tertiary care urban hospital with a clinical phenotype of T2D and DKA at diagnosis, the prevalence of *A*^−^*β*^+^ KPD is 45%. These children share key characteristics previously reported in adults with *A*^*−*^*β*^+^ KPD. Our study highlights the need for increased recognition of pediatric *A*^−^*β*^+^ KPD as a distinct diagnostic entity due to its unique clinical characteristics and course, and implications for prognosis and management. Better understanding of the natural history and pathophysiology of pediatric *A*^−^*β*^+^ KPD should optimize diabetes diagnosis and care, and improve outcomes and quality of life for these children.

## Figures and Tables

**Table 1:** Baseline characteristics of the entire cohort (*n* = 716) and subgroups (those with DKA at diabetes diagnosis (*n* = 56) vs. not (*n* = 660)).

	(*n*)	Entire cohort (*n* = 716)	DKA at diagnosis (*n* = 56)	No DKA at diagnosis (*n* = 660)	*p*-Value (DKA vs. no DKA)
Age^i^ (years), median (Q1–Q3)	716	13.7 (12–15.5)	14.6 (12.5–16.3)	13.6 (11.9–15.4)	0.01
Sex, *n* (%)	716	—	—	—	—
Female	—	449 (63%)	22 (39%)	427 (65%)	<0.001
Male	—	267 (37%)	34 (61%)	233 (35%)	
Race/ethnicity, *n* (%)	702	—	—	—	
Non-Hispanic White	—	64 (9%)	3 (5%)	61 (9%)	
Hispanic	—	411 (59%)	26 (47%)	385 (60%)	0.094
African American/Black	—	205 (29%)	25 (45%)	180 (28%)	
Asian/other	—	22 (3%)	1 (2%)	21 (3%)	
African American/Black, *n* (%)	702	—	—	—	
Yes	—	205 (29%)	25 (45%)	180 (28%)	0.006
No	—	497 (71%)	30 (55%)	467 (72%)	
Tanner stage, *n* (%)	375	—	—	—	0.44
1	—	34 (9%)	1 (4%)	33 (9%)	—
2	—	40 (11%)	4 (17%)	36 (10%)	—
3	—	74 (20%)	5 (21%)	69 (20%)	—
4	—	87 (23%)	8 (33%)	79 (23%)	—
5	—	140 (37%)	6 (25%)	134 (38%)	—
HbA1c^i^ (%), median (Q1–Q3)	632	9.5 (7.2–11.8)	12.2 (11.3–13.1)	9.1 (7.1–11.4)	<0.001
Acanthosis nigricans^i^, *n* (%)	632	580 (92%)	51 (100%)	529 (91%)	0.027
Random C-peptide^i^ (ng/mL), median (Q1–Q3)	470	3 (1.8–4.9)	1.2 (0.8–1.7)	3.2 (2.1–5.2)	<0.001
Glucose^i^ (mg/dL), median (Q1–Q3)	623	234 (158–299)	317 (257–428)	228 (153–294)	<0.001
BMI Percentile^ii^ (%ile), median (Q1–Q3)	707	99 (98–99.5)	99.1 (98.5–99.6)	99 (98–99.5)	0.021
BMI *z*-score^ii^, median (Q1–Q3)	707	2.34 (2.01–2.58)	2.44 (2.17–2.65)	2.33 (2.01–2.57)	0.032
HbA1c at last visit (%), median (Q1–Q3)	717	7.6 (6.2–10.5)	6.3 (5.7–8.3)	7.7 (6.3–10.6)	<0.001

i At diagnosis,

ii at first office visit.

**Table 2:** Multivariable regression model of presence of DKA at diagnosis with age, sex, race (African American vs. not), C-peptide, glucose, HbA1c, and overweight/obesity at diagnosis in children with T2D (*n* = 458).

	Odds ratio	95% Confidence interval	*p*-Value
African American (vs. not African American)	1.6	0.6–3.9	0.34
Male (vs. female)	1.4	0.6–3.4	0.50
Age at diagnosis	1.3	1.1–1.6	0.006
C-peptide at diagnosis	0.2	0.1–0.3	<0.001
Glucose at diagnosis	1.005	1.002–1.009	0.0007
HbA1c at diagnosis	0.85	0.65–1.12	0.25
Overweight or obesity	1.37	0.1–37.53	0.82

**Table 3:** Key clinical and/or biochemical criteria indicative of *β*-cell remission in children with *A*^−^*β*^+^ KPD in those with DKA at diagnosis or within 6 months of diagnosis, no SGLT-2 inhibitor use, negative islet antibodies, and achievement of HbA1c ≤7.5% postdiagnosis.

Eligibility criteria	Number of patients
Insulin discontinuation within 2 years after index DKA event	18
TDD <0.5 units/kg/day	16
Fasting C-peptide >1 ng/mL or random >1.5 ng/mL	4
Insulin discontinuation within 2 years after diagnosis and TDD <0.5 units/kg/day within 6 months	11
TDD <0.5 units/kg/day and fasting C-peptide >1 ng/mL or random >1.5 ng/mL	1
Insulin discontinuation within 2 years after diagnosis and fasting C-peptide >1 ng/mL or random >1.5 ng/mL	1
Patient counts are not exclusive to each category	

**Table 4:** Demographic, biochemical, and clinical characteristics of children with *A*^−^*β*^+^ KPD versus children with DKA who did not meet *A*^*−*^*β*^+^ KPD criteria.

Descriptor	(*n*)	*A*^−^*β*^+^ KPD (*n* = 25)	Did not meet KPD criteria, with DKA (*n* = 31)	*p*-Value
Age at diagnosis, mean ± SD	56	14.9 ± 2	14.1 ± 2.4	0.2
Gender, *n* (%)	56	—	—	0.9
Male	—	16 (64%)	18 (58%)	—
Female	—	9 (36%)	13 (42%)	—
Race/ethnicity, *n* (%)	56	—	—	0.2
White	—	0 (0%)	4 (13%)	—
Hispanic	—	14 (56%)	12 (39%)	—
African American/black	—	10 (40%)	15 (48%)	—
Asian	—	1 (4%)	0 (0%)	—
Other	—	0 (0%)	0 (0%)	—
DKA provoked, *n* (%)	47	—	—	0.079
Yes	—	2 (9%)	8 (32%)	—
No	—	20 (91%)	17 (68%)	—
If provoked DKA, etiology (infection or pancreatitis), *n* (%)	10	—	—	>0.9
Infection	—	1 (50%)	5 (63%)	—
Pancreatitis	—	1 (50%)	3 (38%)	—
Biochemical characteristics, mean ± SD				
C-peptide at diagnosis (ng/mL), mean ± SD	48	1.3 ± 0.7	1.4 ± 1	0.6
Absent islet autoantibodies*, *n* (%)	56	25 (100%)	31 (100%)	—
Glucose^i^ (mg/dL), median (Q1–Q3)	49	305 (254–431)	325 (260–428)	0.8
HbA1c^i^ (%), mean ± SD	49	12.2 ± 1.2	12 ± 1.6	0.6
BMI percentile^ii^ (%), median (Q1–Q3)	56	99.1 (98.3–99.6)	99.1 (99–99.6)	>0.9
BMI z score^ii^, mean ± SD	56	2.3 ± 0.7	2.4 ± 0.5	0.8
Tanner stage^i^	23	—	—	0.3
1	—	1 (13%)	0 (0%)	—
2	—	2 (25%)	2 (13%)	—
3	—	2 (25%)	2 (13%)	—
4	—	1 (13%)	7 (47%)	—
5	—	2 (25%)	4 (27%)	—
HbA1c at last office visit (%), median (Q1–Q3)	54	5.9 (5.7–6.4)	6.5 (5.8–9.8)	**0.038**
Risk factors for DM, *n* (%)				
First degree relative with DM	55	13 (52%)	15 (50%)	>0.9
Acanthosis at diagnosis	51	24 (100%)	27 (100%)	>0.9
Associated obesity comorbidities, *n* (%)				
Hypertension	56	2 (8%)	4 (13%)	0.7
Dyslipidemia	56	22 (88%)	29 (94%)	0.6
Nonalcoholic fatty liver disease	56	2 (8%)	4 (12.9%)	0.7
Polycystic ovarian syndrome (females only)	22	2 (22%)	0 (0%)	0.2

i At diagnosis,

ii at first office visit. Mean +/− SD or median (Q1–Q3) were provided depending on the distribution of the data (normal vs. not).

* Islet antibody test results were not available for confirmation for one patient with *A*^−^*β*^+^ KPD, for whom the parents verbally reported that the test results were consistent with “type 2 diabetes”. Three patients who did not meet criteria for KPD were found to have positive IAA about being on insulin for >200 days, and these positive results were disregarded.

## Data Availability

Data described in the manuscript are available from the corresponding authors upon reasonable request.

## A**ppendix**

### RADIANT Study Group List:

1. Baylor College of Medicine: Ashok Balasubramanyam, M.D., PI^1,2,3,4,5,6,7,8,9^, Maria J. Redondo, M.D., Ph.D., M.P.H., PI^1,3,6,7,8^, Mary Ann Fang, Marcela Astudillo, M.D., Ansley Davis, Dimpi Desai, M.D., Ruchi Gaba, M.D., Nupur Kikani, M.D., Elizabeth Kubota-Mishra, M.S., D.O., Narayan Mulukutla, M.D., Nikalina G O’Brien, Jennifer Posey, M. D., Ph.D.^1,3,7,9^, Stephanie Sisley, M.D.^3^, Mustafa Tosur, M.D.^1^ *Past Staff: Adriana Cardenas, Adrienne Ideozu, Erica Hattery, M.D., M.H.A., Julizza Jimenez, Graciela Montes, Lee-Jun Wong, Ph.D*.
2. Columbia University: Robin Goland, M.D., PI^1,3,4,8^, Wendy Chung, M.D., Ph.D.^7^, Rachelle Gandica, M. D., Rudolph Leibel, M.D.^3^, James Pring.
3. Indiana University: Carmella Evans-Molina, Ph.D., M.D., PI^1,3,7^, Gabriela Monaco, M.D., Anna Neyman, M.D., Zeb Saeed, M.D., Emily Sims, M.D., Maria Spall. *Past Staff: Marimar Hernandez-Perez, Ph.D., Kelly Moors*.
4. Massachusetts General Hospital: Miriam S. Udler, M.D., Ph.D., PI^1,3,7,8^, Jose C. Florez, M.D., Ph.D., PI^1,2,3,4,5,6,7,8,9^, Melissa Calverley, Victoria Chen, Kathy Chu, Sara Cromer, M.D., Aaron Deutsch, M.D., Mariella Faciebene, Evelyn Greaux^6^, Dorit Koren, M.D., Raymond Kreienkamp, M.D., Ph.D., Mary Larkin, R.N., M.S., CDCES, Pam Ricevuto, Amy Sabean, R.N., Jordan Sherwood, M.D., Nop-porn Thangthaeng R.N., Ph.D., CDCES.
5. NorthShore University HealthSystem: Liana K. Billings, M.D., M.M.Sc., PI^1,3^.
6. SUNY Downstate Health Sciences University: Mary Ann Banerji, M.D., PI^1,3^, Necole Brown, Lina Soni, M.D., Lorraine Thomas.
7. University of Chicago: University of Chicago: Louis H. Philipson, M.D., Ph.D., PI^1,2,3,4,5,6,7,8,9^, Siri Atma W. Greeley, M.D., Ph.D., PI^1,3^, Marilyn Arosemena, M.D., Graeme Bell, Ph.D.^3,7^, Colleen Bender, Shanna Banogon, Jui Desai, David Ehrmann, M. D.^3,5,6,7^, Lisa R. Letourneau-Freiberg, M.P.H.^5,6,8^, Rochelle N. Naylor, M.D.^1,3,7,8^, Forough Noohi, Lainie Friedman Ross, M.D., Ph.D.^3,5,6^, Maria Salguero-Bermonth, M.D., Erin Wright.
8. University of Colorado – Denver: Neda Rasouli, M. D., PI^1,3,7,8^, Chelsea Baker, Noosha Farhat, Andrew Her, Courtney King. *Past Staff: Jules Barklow, Rebecca Lorch, Carter Odean, M.S., Gregory Schleis, M.D., Chantal Underko**fl**er*.
9. University of Maryland: Toni I. Pollin, Ph.D., M.S., PI^1,3,8^, Kristin Maloney, M.S., M.G.C.3, Ryan Miller, M.D., Paula Newton, M.D., Maria Eleni Nikita, M. D., Devon Nwaba, M.P.H.^5,6^, Kathleen Palmer, Stephanie Riley M.S., Kristi Silver, M.D., Hilary Whitlatch, M.D.^1^ *Past Staff: Elizabeth Streeten, M.D*.
10. University of Michigan: Elif Oral, M.D., PI^1,3,5,7,8^, Maria Foss de Freitas, M.D., Brigid Gregg, M.D., Seda Grigoryan, M.D., Melda Sonmez Ince, M.D., Adam Neidert, M.S., Carman Richison. *Past Staff: Baris* *A**kinci, M.D., Rita Hench*.
11. University of North Carolina: John Buse, M.D., Ph. D., PI^8^, Jamie Diner, M.S.N.^3,8^, Karthik Edupuganti, Rachael Fraser, Karla Fulghum, Alex Kass^5,6^, Klara Klein, M.D., Ph.D.^1^, Carlos Velez. *Past Staff: M. Sue Kirkman, M.D*.
12. University of Washington: Irl B. Hirsch, M.D., PI^1,3^, Jesica Baran, M.D., Xiaofu Dong, Steven Kahn, M. D.^1,3^, Thanmai Kaleru, Dori Khakpour^6^, Lori Sameshima.
13. Seattle Children’s: Catherine Pihoker, M.D., PI^1,3,5,6,7^, Beth Loots, M.P.H., M.S.W.^6^ *Past Staff: Cisco Pascual*.
14. Vanderbilt University: Kevin Niswender, M.D., Ph. D., PI, Justin Gregory, M.D., M.S.C.I., Alvin Powers, M.D.^1,3^, Andrea Ramirez, M.D., M.S.C.I.^1,3^, Jordan Smith6. *Past Staff: Jennifer Scott*.
15. Washington University: Fumihiko Urano, M.D., Ph.D., PI^1,3,7^, Jing Hughes, M.D., Ph.D.^1,3,7^, Stacy Hurst, Jennifer May, M.D., Janet McGill, M.D., M.A.^1,3,5,6,8^, Stephen Stone, M.D.^1,3,7^
16. Data Coordinating Center – University of South Florida: Jeffrey P. Krischer, Ph.D., PI^1,2,3,4,5,6,7,8,9^, Rajesh Adusumalli, M.S., Bruce Albritton, Analia Aquino, Paul Bransford, Nicholas Cadigan, Laura Gandolfo, Jennifer Garmeson, Joseph Gomes, M.S. Cp.E, Robert Gowing, Christina Karges, M.P.H., Callyn Kirk, M.S.P.H., Sarah Muller^4^, Jean Moris-sette, Hemang M. Parikh, Ph.D.^8^, Francisco Perez-Laras, Cassandra L. Remedios, M.S.^7,8^, Pablo Ruiz, Noah Sulman, Ph.D., Michael Toth, M.S.H.I., Lili Wurmser. *Past Staff: Christopher Eberhard, M.S., Steven Fiske, Brandy Hutchinson, C.I.P., Sidhvi Nekkanti, Rebecca Wood, M.S*.
17. Genetics Core – Broad Institute: Jose C. Florez, M.D., Ph.D., PI^1,2,3,4,5,6,7,8,9^, Ahmed Alkanaq, Ph.D.^3^, MacKenzie Brandes, Nöel Burtt, Jason Flannick, Ph.D.^3^, Phebe Olorunfemi, Miriam S. Udler, M.D., Ph. D.^1,3,7,8^ *Past Staff: Lizz Caulkins*.
18. Genetics Core – Baylor College of Medicine: William Craigen, M.D., Ph.D.^3^, Hongzheng Dai, Ph.D., Shalini Jhangiani, Pengfei Liu, Ph.D.^3^, David Murdock, M.D, Jennifer E. Posey, M.D., Ph.D.^1,3,7,9^, Aniko Sabo, Ph.D.^3,4,7^, Eric Venner, Ph.D.^3^ *Past* *Staff: Lee-Jun Wong, Ph.D*.
19. Central Laboratory – University of Florida: William Winter, M.D.^4^, David Pittman^4^.
20. National Institutes of Diabetes and Digestive and Kidney Diseases: Beena Akolkar, Ph.D.^1,2,3,4,5,6,7,8,9^. *Past Staff: Christine Lee, M.D., M.S*.
21. Other contributors: David J. Carey, Ph.D.^8^, Geisinger Health System. Daniel Hood^8^, Regenstrief Institute. Santica M. Marcovina, Ph.D., Sc.D.^4,5,6,7^, Medpace Reference Laboratories. Christopher B. Newgard, Ph.D.^3,4^, Duke University Medical Center.

#### Committees:

^1^Adjudication, ^2^Ancillary Studies/Data Access, ^3^Discovery, ^4^Laboratory Implementation, ^5^Protocol Implementation, ^6^Protocol Oversight, ^7^Publications and Presentations, ^8^Recruitment and Retention, ^9^Steering.
